# Editorial: Biotransformations by marine microorganisms and their enzymes

**DOI:** 10.3389/fmicb.2023.1192649

**Published:** 2023-04-21

**Authors:** Fabio Parmeggiani, Diego Romano, Josefina Aleu

**Affiliations:** ^1^Dipartimento di Chimica, Materiali e Ingegneria Chimica “Giulio Natta”, Milan, Italy; ^2^Department of Food Environmental and Nutritional Sciences (DeFENS), University of Milan, Milan, Italy; ^3^Department of Organic Chemistry, University of Cádiz, Cádiz, Spain

**Keywords:** biotransformation, biocatalysis, marine microorganism, enzyme, stereoselective synthesis

Biotransformations using microorganisms and isolated enzymes have become an increasingly valuable tool for the production of a wide variety of compounds, from bulk commodities to fine chemicals, specialties and pharmaceuticals. The specific activity and selectivity of the enzymes allow them to perform different chemical reactions with high chemo-, regio- and especially stereoselectivity for the production of enantiomerically pure compounds. A large number of biocatalyzed industrial processes have been already established for the production of an exceptionally broad range of high-added value materials.

In organic synthesis, biocatalytic processes offer a number of advantages over the corresponding chemical methods. The reaction conditions are mild and in the majority of cases do not require the protection of other functional groups. Furthermore, the features governing their regiospecificity differ from those controlling the specificity of traditional chemical catalysts and, indeed, it is possible to achieve biotransformations at centers that are chemically unreactive. Economically, biotransformations are often less expensive and simpler to set up than their chemical counterparts, and the conversions normally proceed under conditions that are regarded as ecologically acceptable. Moreover, enzyme and microorganisms bioprospecting in different habitats could lead to biocatalysts with improved and/or peculiar features.

Thus, the main aim of this Research Topic was to collect original research or review articles focused on the main applications of biocatalysts (whole cells or isolated enzymes) from marine environments for the preparation of bioactive compounds or their precursors, or for the derivatization of natural and synthetic molecules. Within this topic, four articles have been published that complemented our knowledge on biotransformations by marine microorganisms and their enzymes, comprising three original research articles and a review.

Chitosan oligosaccharides (COSs) are derivative products from chitosan or chitin, which have great potential value for use in many areas due to their interesting biological activities. Moreover, COSs are considered as a promising natural antibacterial agent which has been widely applied in food industry and they exhibit many practical advantages such as high water solubility, low viscosity, biodegradability, and biocompatibility. Enzymatic preparation of COSs from chitosan can be carried out by using non-specific enzymes such as protease and cellulase, and specific enzymes such as chitinase and chitosanase. Compared with non-specific enzymes, chitosanase displays higher efficiency and is more suitable for preparation of COSs.

In this Research Topic, Wang et al. characterized a chitosanase (*Sh*Csn46) from marine *Streptomyces hygroscopicus* R1, isolated from shrimp shell waste. The maximum activity and total protein concentration of the recombinant strain *Sh*Csn46 were 2,250 U/ml and 3.98 g/l, respectively. The optimal pH and temperature of purified *Sh*Csn46 were 5.5 and 55°C, respectively. In addition, *Sh*Csn46 exhibited high efficiency to hydrolyze 4% colloidal chitosan to prepare COSs. This work provides a chitosanase with excellent properties for its application in the controllable preparation of COSs.

Further, the same authors (Chen et al.) also prepared and characterized a novel glycoside hydrolase (GH) family 46 chitosanase (*Sl*Csn46) from marine *Streptomyces lydicus* S1. This enzyme was used to controllably produce COSs with different degree of polymerization. The specific activity of purified recombinant *Sl*Csn46 was 1,008.5 U/mg and its optimal temperature and pH were 50°C and 6.0, respectively. Additionally, *Sl*Csn46 can efficiently hydrolyze 2% and 4% colloidal chitosan to prepare COSs. In this work, the potential application of COSs on preservation of tofu was also investigated. The results of this study will provide an efficient chitosanase for controllable preparation of COSs and give some clues on the potential application of COSs on preservation of pre-packaged tofu.

Oceans include very diverse habitats with physical and chemical features that differ considerably from land-based ecosystems. Marine microorganisms are thus adapted to the special conditions of the marine environment and can therefore be an important source of new enzymes with interesting characteristics such as high salinity tolerance, barophilicity and thermostability.

Marine fungi and bacteria account for over 90% of ocean biomass and their diversity is believed to be the result of their ability to adapt to extreme conditions of the marine environment. Their application as biocatalysts in chemical synthesis thus presents a golden opportunity for the development of chemical and pharmaceutical industrial processes, because biotransformations have proven to be a valuable tool in the production of fine chemicals, in particular of enantiomerically pure compounds. Therefore, the screening of new fungal strains with interesting enzymatic activities has become absolutely necessary.

In this Research Topic, Virués-Segovia et al. reviewed the biotransformations carried out by fungi from marine environments from the standpoint of the chemical structure of the substrate, including terpenes, steroids, polyketides, etc. These microorganisms were employed as biocatalysts for different reactions, including hydroxylation, oxidation, reduction, hydrolysis, elimination, rearrangement, cyclization, dehalogenation, etc. Moreover, marine fungi have shown a high potential for the detoxification of pollutants present in wastewater, soils, sediments and solid waste. The review also focused on fungal degradation as an alternative bioprocess for the bioremediation.

Antarctica is known as the continent with the most extreme conditions on Earth. In the last years, the number of studies exploring its microbial biodiversity as an untapped source of extremozymes has steadily grown. Thus, psychrophilic and halophilic microbial communities living in Antarctic Ocean can be an interesting source of new enzymes, which could be used in biotechnological processes. Bisaccia et al. characterized a thermo-, organic solvent- and halo-tolerant laccase (named Ant laccase) isolated from a marine Antarctic bacterium. These authors studied 186 microorganisms isolated from marine biofilms and water samples collected in Terra Nova Bay (Ross Sea, Antarctica) for the identification of new laccase activities. Laccases are copper containing oxidases, which are able to catalyze oxidative coupling or bond cleavage of target compounds with a wide substrate range. After primary screening, some isolates were identified for their ability to oxidize mainly 2,2′-azino-bis (3-ethylbenzothiazoline-6-sulfonic acid) (ABTS). The marine *Halomonas* sp. strain M68 showed the highest activity. This laccase thus becomes a promising candidate for biotechnological and industrial application.

Overall, these four contributions provide the reader with relevant up-to-date insights on the use of enzymes and whole cells from marine ecosystems as biocatalysts.

## Author contributions

All authors listed have made a substantial, direct, and intellectual contribution to the work and approved it for publication.

